# A Thematic Analysis Investigating the Impact of Positive Behavioral Support Training on the Lives of Service Providers: “It Makes You Think Differently”

**DOI:** 10.3389/fpsyg.2019.02408

**Published:** 2019-10-29

**Authors:** R. Stephen Walsh, Brian McClean, Nancy Doyle, Suzanne Ryan, Sammy-Jo Scarborough-Lang, Anna Rishton, Neil Dagnall

**Affiliations:** ^1^Department of Psychology, Manchester Metropolitan University, Manchester, United Kingdom; ^2^Acquired Brain Injury Ireland, Co., Offaly, Ireland; ^3^Future Directions CIC, Greater Manchester, United Kingdom

**Keywords:** positive behavioral support (PBS), training, thematic analysis, staff experience, challenging behavior

## Abstract

Positive behavioral support (PBS) employs applied behavioral analysis to enhance the quality of life of people who behave in challenging ways. PBS builds on the straightforward and intuitively appealing notion that if people know how to control their environments, they will have less need to behave in challenging ways. Accordingly, PBS focuses on the perspective of those who have behavioral issues, and assesses success *via* reduction in incidences of challenging behaviors. The qualitative research presented in this report approaches PBS from a different viewpoint and, using thematic analysis, considers the impact of PBS training on the lived experience of staff who deliver services. Thirteen support staff who work for a company supplying social care and supported living services for people with learning disabilities and complex needs in the northwest of England took part. Analysis of interviews identified five major themes. These were: (1) training: enjoyable and useful; (2) widening of perspective: different ways of thinking; (3) increased competence: better outcomes; (4) spill over into private lives: increased tolerance in relationships; and (5) reflecting on practice and moving to a holistic view: “I am aware that people…are not just being naughty.” These themes evidenced personal growth on the part of service providers receiving training. Explicitly, they demonstrated that greater awareness of PBS equipped recipients with an appropriate set of values, and the technical knowledge required to realize them.

## Introduction

Positive behavioral support (PBS) is the application of applied behavioral analysis (ABA) (Baer et al., 1968; Allen et al., 2005). Hence, researchers define PBS as “the scientific study of behavior change, using the principles of behavior, to evoke or elicit a targeted behavioral change” (Furman and Lepper, 2018, p. 104) in people with challenging behaviors. Its primary goal is to enhance the quality of life of people who behave in challenging ways (LaVigna and Willis, 2005). Hence, a key focus is individual environments. These can be adapted so that challenging behavior is less necessary. Particularly, through the acquisition of more socially effective alternative behaviors, where people are motivated to replace inappropriate, stigmatizing, or destructive ways of responding (LaVigna and Willis, 2005).

The core idea that PBS builds on is straightforward and intuitively appealing: if people know how to control their environments, they will have less need to behave in challenging ways (Hassiotis et al., 2014). PBS imparts this knowledge *via* instruction, and considers the efficacy of training from the point of view of quality of life of the person behaving in challenging ways, and in terms of reduction in incidences of challenging behaviors (e.g., McClean et al., 2005; Walsh et al., 2018). In this context, behaving in challenging ways refers to “Culturally abnormal behavior of such intensity, frequency and duration that may put the person or others physical safety in jeopardy or seriously limit the use of community activities” (Emerson, 2001, p. 7).

PBS is an important treatment framework in the field of learning disability (Hassiotis et al., 2014; Gore et al., 2019). PBS is also a useful approach for those working with teenagers and young adolescents, groups for whom challenging behaviors can have a serious impact on the services that they receive (Bohanon et al., 2006). An important pathway through which challenging behaviors can negatively affect service delivery is *via* the staff who deliver the services. As such, staff welfare is of fundamental importance (Williams and Glisson, 2013).

Traditionally, expert opinion rather than user-perceptions has driven behavioral interventions (LaVigna and Willis, 2005). In contrast, some theorists switch the focus of PBS to person-focused training of stakeholders (i.e., McClean et al., 2005; Grey and McClean, 2007), for example, the person presenting with challenging behavior, their families, and service providers. From this perspective, those people impacted by the behavior are paramount – rather than passive recipients of instruction from an “expert.” Stakeholders are active participants in assessment, determining intervention strategies, evaluation of these strategies, and thinking about what outcomes might influence service user’s quality of life (World Health Organization, 2006). In working environments where resources are scarce, even when the benefits of staff training appear evident, justifying incumbent costs can prove difficult (Dench, 2005). In this context, acknowledging the central role of stakeholders with regard to the implementation of PBS (Dench, 2005), it is vital that researchers consider the impact of PBS training on those who deliver the support.

The present study explored how training in PBS affected the lived experience of those receiving training. Thus, it adopted the view that training is a “collaborative project” to which people commit themselves and meaning making is understood as residing between people rather than within individuals. Previous research in PBS has tended to focus on the impact of PBS on incidents of challenging behavior (Walsh et al., 2018). An important, and yet unanswered, question was whether those trained to deliver PBS, with a view to improving the lives of others, experienced any benefit from such training in their own lives.

## Materials and Methods

### Approach to Data Collection

This study, consistent with Braun and Clarke (2006), used thematic analysis in an open-ended way, to investigate how participants experienced the impact of PBS training in both their professional and private lives. The researchers employed a purposive sampling strategy whereby they engaged with a service provider who delivers PBS training to staff as part of their on-going professional development.

### Ethical Protocol

The study received full ethical approval from the Manchester Metropolitan University (MMU) ethics committee. All participants provided written informed consent. The study brief informed them that they were free to withdraw at any time, should they wish to do so. Participants consented to the recording of interviews, which were subsequently anonymized and transcribed. Interviews were stored on a password-protected (encrypted) computer, which housed all data.

### Interview Process

Participant interviews occurred in their place of work on a prearranged and mutually agreed day. Interviews were semi-structured; a guide provided a loose structure within which to explore the topics of interest. The central question was “what impact has PBS training in the lives of those who receive it?” Where appropriate, the interviewer prompted participants to expand on relevant and interesting responses.

### Participants

Purposeful sampling is a widely used technique in qualitative research whereby those cases most likely to be information-rich on the point of interest are selected in order to effectively use limited resources (Patton, 2002). To this end, only staff who had received PBS training were recruited. All staff approached for participation were over the age of 18 and all were permanent employees. The researchers sent an email to potential staff participants requesting volunteers to take part in interviews with regard to their experience of PBS training. A similar advertisement appeared also notice boards in common areas. Respondents participated without incentives. Thirteen participants were interviewed for the purpose of this study. As the goal of the study was to gain a depth of understanding on the point of interest (i.e., participants’ experience of PBS training), through the recruitment of a homogenous^1^ sample, data such as mean age etc. are not reported as it might convey the unwarranted impression of generalizability and quantitative robustness.

The service provider delivering the training in PBS was a Community Interest Company providing social care and supported living services for people with learning disabilities and complex needs in the northwest of England. The company is a value-based, high-quality social care provider whose goal is to enable meaningful living among clients. The service provider works with people across a range of environments to provide a continuum of support ranging from a few hours of home care to 24/7 supported living services, and higher levels of support in residential services. All clients are over the age of 16. Supported individuals may have a mental health diagnosis, autism, complex health, profound multiple disabilities, be young people in transition, or have a learning disability, forensic history, acquired brain injury, or dementia.

The company has embedded PBS within service provision. In addition to training staff in PBS, the company has a PBS lead who ensures that staff training remains current. The company also has trained active support champions who facilitate the application of learning to practice.

All staff receive 3-day induction training, which includes consideration of autism, communication, and positive behavioral support. All managers have a level 2 training day covering PBS key components, values, theory, and process. Managers learn also how to develop individual PBS plans, which include functional assessment. PBS plans are evidence based, with 80% of the plan being proactive in order to ensure the achievement of good client outcomes. Training emphasizes that all behavior is for a reason.

All staff receive active support training. This outlines that participation and engagement represent meaningful activities that anybody can engage. The service provider has PBS champions that support staff practically in their job, ensuring that active support is embedded as part of the culture. This role ensures the people supported are empowered in their environment regardless of their ability and actively participate and engage in every part of their life.

Additionally, the company distributes Monthly Newsletters to staff as an additional teaching aid. These share good news stories including information on telecare and other technology designed to give people more choice and control over their lives. Alongside this, training facilitators provide further specialist training. Finally, teams use a training DVD produced by service users to embed staff training.

### Data Analysis

This study used thematic analysis (Braun and Clarke, 2006). This required the transcription of interview recordings and followed coding stages. Initially, the authors read and re-read transcripts in order to identify potential themes, which they then forwarded to the lead author. The second level of analysis involved both the first and last authors reviewing these initial codes. They considered particularly how to retain the diversity of the initial codes, while producing overarching elements, higher level sub-themes. The research question, the impact of PBS training in the lives of participants, informed this process. At the third stage, analysis conducted by the first and last authors identified quotes that were congruent with the overarching themes. Next, the authors reviewed themes prior to defining and naming them. Finally, once themes were finalized, by the first and last authors, the write-up of the report began.

## Results

The analysis produced five themes.

### Training: Enjoyable and Useful

Almost all participants reported that the training that they received was both enjoyable and useful. Illustrative examples appear below.

One participant stated:

MOLLY: *I really enjoyed the course and everything and it did make me understand a little bit more.*

Participants highlight the enjoyment that they derive from their PBS training course and they explicitly tie this enjoyment to their capacity to internalize it.

ANN: *Just listen. Enjoy it. You’ll take something from it even if you do not realize that you do. You think back to what it actually was and you realize that you did take a lot from it.*

### Widening of Perspective: “Different Ways of Thinking”

In their accounts, most participants highlighted how their perspectives broadened following PBS training.

CATH: “*This is a different way of thinking and getting staff to think differently.”*

Participants gave examples of changes in their thinking such as exploring why a person might be upset (Molly), looking for triggers (Maria) being more aware of the possibilities for support in a given moment (p1, Ann) and many participants noted a widening of perspective:

ZOE: “*I see it differently now when somebody is getting anxious. We only see people for a short time. Not that we would leave anybody anxious but it makes you think differently. Thinking outside the box.”*

### “Increased Competence: Better Outcomes”

A third theme is perceptions of increased competence, and the role of increased competence in promoting better client outcomes.

HEATHER: “*I know what to do in a certain situation whereas some people who hadn’t had it wouldn’t know what to do.”*

Participants noted more detailed understanding of triggers (Freya) and better ability to read the communicative intent of clients (Molly). One participant reported that clients “*don’t get to that agitated point like you can prevent it from happening because you know that the reason they are, like, representing the challenging behavior is that they want something or something’s annoyed them.”* (Ann).

Other participants reported better outcomes for clients as a result:

ROBIN: “*One person I support has been with [service provider] for 11 years and has always been supported 1:1. I would say roughly he was having 3 incidents a week. (Since PBS was introduced) he has been going out on his own now for 2 months on local walks, walking 2 miles. I don’t think we have had incidents in 2 months. PBS has made it easier, the paperwork side, trying to show staff they wouldn’t be at fault. The staff were scared. I was 5, 6 years ago, but when you come to think about it, it’s better for the person.”*

### Spill Over Into Private Lives: Increased Tolerance in Relationships

Many participants describe the impact PBS training made in their lives beyond the workplace. Participants say they can apply the principles directly with their family members.

MARIA: “*At home, my children … will come to talk to me, they need attention, and I say ‘I’m talking on the phone, you have to wait’, but now, instead of shouting at them I give them attention but not stop, ask them to write it and come to me, then I will tell you what to do. ... To know that there is a reason for any behavior, and how to handle it.”*

MAUREEN: “*It (PBS training) has impacted on outside as my partner has high anxiety levels. I have looked at triggers, I have tried to reduce his anxieties using PBS and the techniques.”*

ANN: *My sister, my middle sister, she’s got learning disabilities. So, like, cos it is simple things like you have got to recognize that most of the challenging behavior is because they are trying to communicate something. Even that feel good factor, or they are just doing it to release some stimulation. But you just need to realize that there is a reason behind all of it, is not there? It’s not just the naughty child, or whatever people use as an excuse.*

In terms of indirect impact on participants’ private lives, they spoke about being less judgmental and more effective in their close personal relationships after PBS training:

MARIA: *I apply it in my everyday life, especially to be non-judgemental.*

ZOE: *Yes, because when I had the training we talked about when you have a bad day how you would react, and how your partner would (react) to you…(as a result) I tried something different.*

### Deeper Understanding of People

Participants reflected on a theme that their philosophy of people had changed. For example, they noted a different attitude to behaviors outside work.

HEATHER: “*I’m aware of people when I’m out (outside of work) that they have got behavioral problems and they’re not just being naughty.”*

Commensurate with this change in philosophy is a different or deeper understanding of how people need to be treated.

ANN: *It’s encouragement, rather than punishment. That’s what I have taken from it, less telling off and more understanding and encouragement*.

## Discussion

Traditionally, expert opinion rather than user-perceptions has driven behavioral interventions (LaVigna and Willis, 2005). Training in PBS is important because it switches focus to the training of stakeholders. The impact of PBS training on staff has been under researched (Dench, 2005). Dench (2005) argues that organizational best practice means that personal development should link to institutional goals and that training evaluation should include qualitative perspectives. It was therefore vital to consider the impact training in PBS has on staff from a qualitative point of view. Staff are key stakeholders and active participants in assessment, determining intervention strategies, evaluation of these strategies, and thinking about what outcomes might affect service user’s quality of life. Vygotsky regarded learning as the ingrowing of lived experience into personal meaning, an outside-in approach (Frawley, 1997). This outside-in perspective lends itself readily to a consideration of how being trained in PBS influences the lived experience of those receiving training. Our results show, within the cohort sampled, that the impact on individuals was overwhelmingly positive.

Specifically, the participants in our research reported that PBS training was enjoyable. This was the case at both emotional and cognitive levels, where training represented both participant’s experience as well as its environmental context (Wankel, 1993). Thus, consistent with Dench (2005), enjoyment constituted a framework for further embedding training content. Participants also described a widening of perspective – this experience is consistent with the person-based focus advocated by PBS. Moreover, a widening perspective is congruent with the approach advocated by educationalists who build on Vygotsky’s legacy to move education and training away from a focus on test performance to addressing individual capabilities in a grounded and creative manner (e.g., Craft et al., 2008).

PBS training is perhaps best conceived as a “collaborative project”, an aggregate of actions that are directed toward an aim. However, at the same time, a project is not equated with its aim, “a unit of educative work in which the most prominent feature was some form of positive and concrete achievement”. Participant 8 spoke about a client who had shifted from three incidents of challenging behavior per week pre PBS training to a position where the client is now going out, unaccompanied, for 2-mile walks.

When a project manages to achieve relatively permanent changes in the social practices of a community, it evolves from being a social movement into an institution. This fits well with Dench (2005), who argues that best practice in training leads to an integration between human resource development and management policies and processes. There was evidence that this was indeed the case with our participants. For example, one participant expressed a desire to have all staff undergo PBS training at induction, and for the implementation of annual refresher training. Participants described also how the benefits of PBS training have “spilled over” into their private lives. Specifically, people who received training described how their marital, sibling, and parental relationships improved. Increased self-efficacy (Bandura, 1997) is a key factor in how individuals’ personal development opportunities link to specified organizational goals.

Our final theme pertained to the deeper understanding of people whom participants describe because of their training. Several of those interviewed made reference to moving beyond considerations based around ideas of people “being naughty” and reflected on a move to a more holistic approach, where their attitude was significantly less judgmental, efficacious, and increasingly tolerant [e.g., “I apply it in my everyday life, especially to be non-judgemental. To know that there is a reason for any behavior and how to handle it” (Gore et al., 2013)].

These themes all appear to link with the concept of perceived control, and perceptions of personal control are key to managing both work and home environments in positive ways. According to Bandura (1997), knowing how to develop and exercise efficacy is a useful basis for well-being enhancement. Social norms convey standards of conduct, when participants adopt these, as they clearly did in the present study, a self-regulatory system consistent with these standards emerges (Bandura, 1986). From an organizational perspective, this last point is key. Training staff in PBS produced elevated perceptions of increased control. Such perceived control is of benefit to both the individuals and their organization (Bandura, 1997). In the current climate, where resources are scarce, all expenditure, including that on training, must, and should, be fully justified. The results of the current study clearly suggest that training staff in PBS offers benefits at the level of service provision as well as at both personal and corporate levels.

### Limitations and Future Research

The present paper identified the importance of perceived control. This is an important finding, which requires cautious interpretation because researchers define perceived control in different ways (Chipperfield et al., 2012). Some employ the classic definition, which refers to beliefs about influence. Other theorists prefer a liberal interpretation that denotes perceived control as a psychological state of control. The emphasis with this delineation is whether individuals feels “in or out of” control. The former conceptualization focuses on specific outcomes, whereas the latter is broad and general. This distinction is one which might usefully be considered in future studies. Of importance will be investigating the extent to which vocational training increases perceived control across life domains. The implicit assumption within the present paper was that the benefits were broad (extended beyond practice to family and relationships generally). However, this is difficult to establish without further consideration of different contexts/situations.

In addition, other factors limit the generalizability of findings presented in this report. Specifically, conclusions derive from a small-scale qualitative study centering on a single service provider. Consequently, it is unclear whether the observed benefits extend across service providers and organizations. This is something that subsequent studies should investigate. This could include evaluation of similar service providers, service providers generally, and extend eventually to consider the benefits of occupational training. Clearly, if research evidences that training benefits both clients and practitioners, this from a vocational and practical perspective indicates that it necessitates resourcing.

Noting the limited scope of the current study, further work could examine the outcomes using larger samples and relevant objective psychometric measures, for example, scales assessing perceived control, self-efficacy, and well-being. Longitudinal analysis might establish causal relationships and reveal whether benefits sustained over time. Furthermore, larger samples allow the testing of predictive relationships and the development of models.

Acknowledging these limitations, readers should best consider the study findings in terms of transferability rather than in terms of generalizability. It is also necessary to put on the record the specific interests of the author and research team, which may have inadvertently influenced both the content and findings presented in this report. In particular, their interest and in the service provider and the accompanying community psychology project.

## Conclusion

PBS training equips those who receive it with a set of values, as well as the technical knowledge required to realize those values (Walsh et al., 2018). An important goal of PBS training, in common with training in all fields, is that the training is internalized by those who receive it in order to widen their perspectives and contribute positively to wider institutional and societal well-being. There is evidence that the training has been internalized, in Vygotsky’s sense of the inter-psychological becoming the intra-psychological. Staff understand themselves as having benefitted from PBS training and they believe this benefit extending beyond their professional lives. This perceived gain speaks to the importance of training. Particularly, it evidences the positive impact it has on both the lives of those who receive it as well as on the lives of those around them. As such, PBS training fits with a holistic approach to service provision that is mindful of the importance of caregiver well-being in addition to client well-being (see MacDonald and McGill, 2013). In sum, what the themes identified in this research evidence, and share, is growth on the part of those who received training in PBS.

## Ethics Statement

The study received full ethical approval from MMU ethics committee. Participants were advised that they were free to withdraw at any time, should they wish to do so. All interviews were recorded with the permission of participants and they were later anonymized and transcribed. Anonymized interviews were stored on a password-protected computer for later analysis.

## Author Contributions

NDa, S-JS-L, and SR collected data. All authors participated in thematic analysis. RW, BM, and NDa wrote up the final report with feedback and contribution from all authors.

### Conflict of Interest

The authors declare that the research was conducted in the absence of any commercial or financial relationships that could be construed as a potential conflict of interest.

## Footnotes

^1^Homogenous on the point of interest – PBS training.
